# Cognitive impairment in bipolar disorder Neuroprogression or behavioral variant frontotemporal dementia?

**DOI:** 10.1590/1980-57642018dn13-040016

**Published:** 2019

**Authors:** Saulo Queiroz Borges, Thiago Xavier Corrêa, Isabela Oliveira Azevedo Trindade, Rivadávio Fernandes Batista Amorim, Maria Alice de Vilhena Toledo

**Affiliations:** 1 MD, Universidade de Brasília (UnB), Hospital Universitário de Brasília (HUB), Brasília, DF, Brazil.; 2 Ft, MSc, Universidade de Brasília (UnB), Hospital Universitário de Brasília (HUB), Brasília, DF, Brazil.; 3 MSc, PhD, Universidade de Brasília (UnB), Hospital Universitário de Brasília (HUB).; 4 MD, MSc, PhD, Universidade de Brasília (UnB), Hospital Universitário de Brasília (HUB), Brasília, DF, Brazil.

**Keywords:** bipolar disorder, frontotemporal dementia, geriatric neuropsychiatry, neuroprogression, transtorno bipolar, demência frontotemporal, neuropsiquiatria geriátrica, neuroprogressão

## Abstract

Patients with Bipolar Disorder (BD) usually display cognitive deficits with aging. However, the correlation between BD and dementia syndromes is inconclusive, despite the similarity with behavioral variant frontotemporal dementia. We report a 78-year-old female patient who had bipolar type 1 disorder since adolescence. Her symptoms ranged from apathy to psychotic mania. She had had three hospitalizations, and since her last stay 10 years ago, her symptoms had remained stable. However, in the past 2 years, she displayed different symptoms, such as irritability manifested as verbal and physical aggression, cognitive impairment, repetitive pattern of behavior, perambulation, persecutory delusions, disorientation, and hyporexia. Treatment with anticholinesterases or mood stabilizers promoted no improvement. She scored 17/30 points on the Mini-Mental State Examination. Neuropsychological assessment suggested deficits in executive function, attention, and memory. Neuroimaging tests revealed frontotemporal degeneration and hypoperfusion. Diagnostic and therapeutic approaches for this type of patient represent a significant challenge for clinicians.

## RESUMO

Pacientes com Transtorno Bipolar (TB) costumam apresentardéficits cognitivosao envelhecer. No entanto, a correlação com síndromes demenciais é inconclusiva, apesar da similaridade com a variante comportamental da demência frontotemporal (bvFTD). Nósrelatamos uma paciente de 78 anos de idade com TB tipo 1 desde a adolescência. Seus sintomas variavam de apatia a mania psicótica. A paciente passou por 3 internações, sendo a última há 10 anos, seguida de estabilização clínica. No entanto, nos últimos 2 anos, ela apresentou sintomas diferentes, como irritabilidade expressada por agressões verbal e física, comprometimento cognitivo, padrão repetitivo de comportamento, perambulação, delírios persecutórios, desorientação e hiporexia. O tratamento com anticolinesterásicos ou estabilizadores de humor não revelou melhora. Apresentou 17/30 pontos no miniexame do estado mental, a avaliação neuropsicológica sugeriu déficit de função executiva, atenção e memória. Os exames de neuroimagem demonstraram atrofia e hipoperfusão fronto-temporal. Abordagens diagnósticas e terapêuticas para este tipo de paciente representam um desafio significativo para os clínicos.

Cognitive impairment has a close relationship with mental disorders. In this scenario, Bipolar Disorder (BD) presents the highest risk for the development of dementia syndromes when compared to other clinical diseases.1 Cognitive impairments in the course of BD affect mainly memory, attention, language, and executive functions, even during the euthymia stage.2 Despite the similarities with behavioral variant frontotemporal dementia (bvFTD), attempts to correlate BD with dementia syndromes have proved inconclusive.3 Although some studies reveal stability of cognitive impairment,4 others show cognitive impairment and neuroprogression to dementia.5

It is worth mentioning that doubts concerning the differentiation between the cognitive impairment typi cal in older patients with bipolar I disorder (BDI) and that seen in other dementias pose diagnostic challenges and can lead to treatment failures. For instance, the use of anticholinesterasics in patients with ith BDI may not be appropriate, with some cases of manic-switch reported.^6^ In this context, the present study aims to describe the observations of a patient who clearly displayed neuropsychiatric symptoms and the possibility of neuroprogression in BDI. Based on this report, we intend to draw neuropsychological inferences and demonstrate the need for a better understanding on evolution of bipolar patients. 

## CASE REPORT

A 78-year-old woman was admitted to a university clinic with complaints of cognitive impairment associated with functional deterioration and worsening behavior. At admission, she was totally dependent according to the Lawton Instrumental Activities of Daily Living Scale (IADLs) (0/9 points)^7^ and semi-dependent according to the Katz index of independence in Activities of Daily Living (Katz ADL) (4/6),^8^ requiring assistance for bathing and tooth brushing. The patient had received 4 years of education and was diagnosed with BDI at age 16. She had been given galantamine 16 mg/day because of suspected Alzheimer’s disease (AD). Despite the treatment, the patient displayed worsening hyporexia, psychomotor agitation, and no clinically significant improvements in cognition, behavior, or functioning. 

She had had three hospitalizations and the symptoms ranged from apathy to euphoria with psychotic symptoms. The last hospitalization occurred 10 years before referral. After hospital discharge, the patient remained in euthymia for 6 years and was treated with oxcarbazepine at a dose of 600 mg/day, bromazepam 3 mg/day, and risperidone 1 mg/day. However, the patient reported progressive neuropsychiatric alterations in the last 2 years with different characteristics, such as impulsivity and irritability manifesting as verbal and physical aggression, short-term memory loss, repetitive pattern of behavior, perambulation, persecutory delusions, disorientation, and hyporexia. She also presented with occupational impairment and loss of functionality, forcing her to take leave from her work activities.

**Figure 1:**
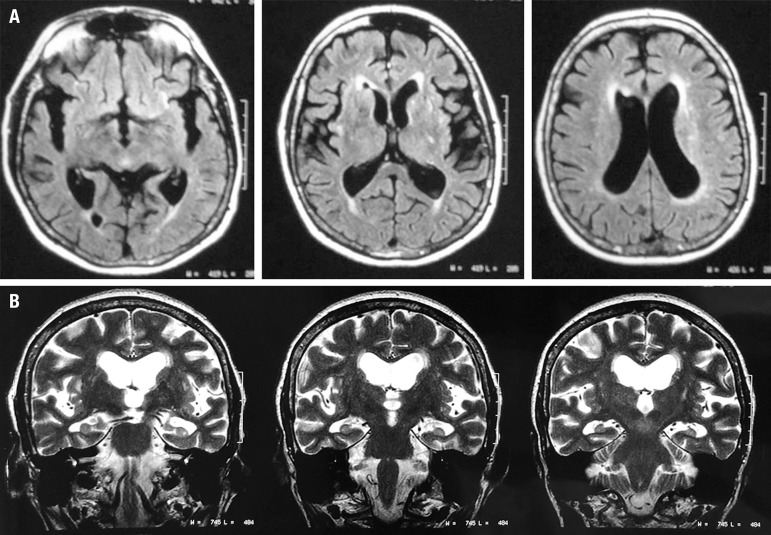
**[A]** Magnetic Resonance Imaging (MRI)* **[B]** Magnetic Resonance Imaging (MRI)**. *Axial Flair Brain MRI shows cortical atrophy with frontotemporal predominance and abnormal signal intensity in white matter suggestive of incipient ischemic microangiopathy. **Coronary slices show global atrophy with hippocampal atrophy.

**Figure 2:**
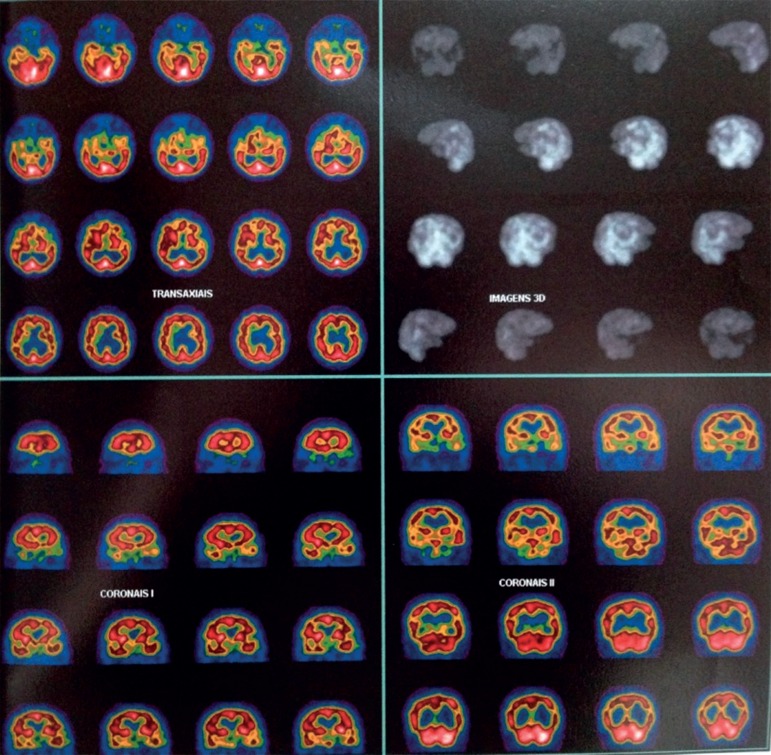
Single-Photon Emission Computed Tomography (SPECT). Image showing moderate-to-severe hypoperfusion/hypoactivation in regions of the bilateral frontal and temporal cortices.

Neuropsychological evaluation revealed executive function, language, memory and attention deficits, with considerable frontal lobe involvement (Table 1). In her recent neuroimaging studies, magnetic resonance imaging (MRI) of the brain showed brain atrophy with frontotemporal predominance and incipient ischemic microangiopathy (Figure 1). Single-photon emission computed tomography (SPECT) revealed moderate-to-severe bilateral frontotemporal hypoperfusion/activation (Figure 2) The patient had a long history of medication use, which included lithium carbonate, sodium valproate, olanzapine, sertraline, escitalopram, and trazodone. She presented with systemic arterial hypertension and hypothyroidism. She reported no history of alcoholism, smoking, or illicit drug use. Two sisters had psychiatric disorders and the mother had an unspecified dementia syndrome.

**Table 1:** Neuropsychological test results.

Functions evaluated			Very low (<3 SD)	Low (-3 SD)	Low Average (-2 SD)	Average (± 1 SD)	High Average (+2 SD)	High (+3 SD)	Very High (>4 SD)
IQ		General IQ	●	●	●				
Attention and Executive Function		Auditory/verbal attention amplitude	●	●					
		Auditory/verbal working memory	●	●					
		Copy of alternating drawings	●	●					
Visuo-Spatial Organization		Copy of simple geometric stimulus	●	●	●	●			
		Drawing of simple figures	●	●	●	●			
		Clock drawing	●	●					
		Perspective figure copy	●	●					
Language		Semantic verbal fluency	●	●					
		Naming by visual confrontation	●	●	●				
		Understanding simple verbal commands	●	●					
		Abstract verbal thinking	●	●					
Visual Functions		Visual gnosis	●	●	●	●			
Motor Functions		Alternate manual movements	●	●					
Memory	Verbal	Immediate verbal episodic memory	●	●					
		Delayed verbal episodic memory	●	●					
		Cued verbal learning	●	●					
		Verbal learning - delayed recall	●	●					
		Verbal recognition (Errors = 8)	●	●					
		Associative memory	●	●					
	Visual	Immediate visual memory	●	●					
		Delayed visual memory	●	●					

Clinical examination revealed signs of parkinsonism, aggressiveness, apathy, speech impairment, depressed mood, and facial hypomimia. On cognitive assessment, the patient scored 17/30 points in the Mini-Mental State Examination (MMSE) and 5 points in the verbal fluency test using the animal category. Laboratory and serology tests for secondary dementia screening were all within the normal range. In addition, the diagnosis of *delirium* was excluded based on complementary exams and also considering that the disease is characterized by acute symptoms with fluctuating clinical course.

In this context, we hypothesized the presence of a non-Alzheimer’s dementia syndrome with possible bvFTD or cognitive impairment as a result of neuroprogression in BDI. We opted to suspend galantamine, withdraw risperidone and continue with quetiapine. Although the patient progressed with partial improvement of psychomotor agitation, she showed gradual worsening of apathy and aphasia, with progressive cognitive/functional losses. The patient died 4 years later from pulmonary infectious complications.

## DISCUSSION

Although our patient revealed a profile and clinical evolution similar to those described in neuroprogression in BD,^9^ the association with other dementia syndromes, especially bvFTD, could not be excluded. A systematic review describing cognitive impairment in bipolar patients has been published, especially addressing executive function disorder.^10^ It suggested that bipolar patients have a reduced cerebral reserve capacity, demonstrated by worsening cognitive impairment throughout the lifespan.^10^ The type of cognitive impairment observed in BD patients may have a clinical, neuropsychological and imaging presentation that resembles bvFTD.^11^ Clinical observations report neuroprogression to dementia in patients with BD over a 30-year period.^12^ Some recent studies have suggested an etiopathogenic association between BD and a specific dementia syndrome, similar to bvFTD.^12^

In the present case, we observed a change in our patient’s patterns of behavior when compared to the symptoms previously presented, but occurring at a later age. These changes failed to respond adequately to the use of mood stabilizers or anticholinesterasics, evolving with behavioral worsening, and progressive cognitive and functional impairment. Based on the current diagnostic criteria for bvFTD,^13^ and on evidence showing that age has little influence on manic psychopathology,^14^ it is possible to affirm that our patient had probable bvFTD, although we understand that BD could cause underlying cognitive impairments. Even though bipolar patients have an increased risk of cognitive impairment associated with age-related pathologies,^1^ atypical Alzheimer’s dementia was not considered as a differential diagnosis based on initial behavioral worsening, therapeutic response and neuroimaging findings. 

The literature shows impact on the psychosocial functioning of these patients mainly due to mood state and cognition, which may remain even after the acute phases of the disease.^2^

Factors linked to BD that may influence cognition include number of mood or psychotic episodes, hospitalizations, age at onset, duration of illness, polypharmacy, and presence of clinical comorbidity.^10^ Our patient had a long history of BDI, with past psychiatric hospitalizations, psychotic symptoms, and use of various psychotropic medications.

Elderly patients with BD had worse performance on psychomotor speed, attention, memory, verbal fluency, and executive functions, as well as worse psychosocial functioning, independent of current mood state or iatrogenic effects of psychotropic drugs.^15^^,^ 16 Neuropsychological assessment suggested an impairment pattern that could be present both in patients with BDI and dementia syndromes.^2^ Thus, establishing a differential diagnosis of bvFTD in a bipolar patient becomes a challenge, and we could not rule out a causal association between the two entities.

There is weak evidence that cortical atrophy and white-matter vascular lesions are more common in older people with BD than in normal controls, where it remains uncertain whether this finding is attributed to the psychopathological process or to secondary factors.^17^ We performed neuroimaging analysis to evaluate the differential diagnosis considering that the patient exhibited behavioral and cognitive changes suggestive of bvFTD. In our patient, functional and structural neuroimaging studies revealed signs of hypofunction and atrophy of the frontotemporal region, as demonstrated in scientific literature establishing a possible association between frontal and temporal circuit dysfunction and certain cognitive impairments.^18^ A follow-up study established an association between BDI severity, memory loss, and reduction of gray matter volume in the medial temporal cortex.^19^ This information may be strongly associated with frontal and social deficits exhibited by individuals with BD. The vascular hypothesis for the impairments found in our patient would be less likely given the minor subcortical vascular damage observed, relatively common in patients from this age group.^20^

Although signs of memory deficits have been reported as commencing together with the patient’s functional decline, disagreement between the onset of objective memory impairments and the observation of the symptoms by third-parties is not uncommon, usually because these individuals are unaware of dementia syndromes. A possible hypothesis could be the mistaken notion that memory loss and functional decline are an inevitable part of aging or a refusal to accept a decline in the functional abilities of relatives with dementia.^21^ Although memory is only affected in more advanced stages of FTD, the initial picture of apathy, irritability, and impairment in self-control, self-care, and body hygiene revealed by our patient fits into the clinical manifestations typical of FTD patients. In the case of FTD, behavioral changes tend to be more prominent in the early stages, with preservation of visuospatial functions, spatial orientation, and memory, contrasting with milder changes in behavior, less self-neglect and emotional blunting, and a longer course related to a possible distinct dementia in BD.^13^^,^ 22

The concept of clinical heterogeneity is present in both pathologies, representing a difficulty in establishing precise diagnostic criteria, particularly in bvFTD, whose criteria have been the subject of discussion and proposals for revision due to the expansion of the functional, genetic, and biochemical knowledge.^23^ Hypotheses related to neuroprogression and neurodegeneration may justify the cognitive impairments presented by patients with BD, probably due to a combination of early genetic factors, environmental risk factors, medical comorbidities, iatrogenic effects, and aging itself.^23^^,^ 24 Glial cell density reductions demonstrated in postmortem studies in patients with BD reveal that their brains may be more vulnerable to toxic, metabolic, and ischemic insults during adult life.^25^

In view of the information presented here, it is pertinent to infer the need for further prospective studies, especially cohort studies associated with postmortem pathological analysis involving patients with BDI, to better clarify the biological plausibility of cognitive impairment, which might be a multifactorial entity with different evolution patterns from those observed in bvFTD. Another important aspect is the establishment of new protocols for neuropsychological assessment and of nosological classification criteria with the use of neuroimaging for diagnostic elucidation.
